# A perioperative nursing care protocol for patients with spinal muscular atrophy (SMA) type II or type III undergoing spinal surgery: a 4-year experience in 24 patients

**DOI:** 10.1186/s13023-025-03718-z

**Published:** 2025-05-19

**Authors:** Gaoyang Li, Kexin Xu, Di Liu, Nan Wu, Terry Jianguo Zhang, Yaping Chen

**Affiliations:** 1 Department of Orthopedic Surgery, State Key Laboratory of Complex Severe and Rare Diseases, Peking Union Medical College and Chinese Academy of Medical Sciences, Peking Union Medical College Hospital, No. 1 Shuaifuyuan, Beijing, 100730 China; 2 Beijing Key Laboratory of Big Data Innovation and Application for Skeletal Health Medical Care, Beijing, China; 3 Key Laboratory of Big Data for Spinal Deformities, Chinese Academy of Medical Sciences, Beijing, China

**Keywords:** Spinal muscular atrophy, Nursing care, Disease management, Muscle weakness, Orthopedic procedures, Perioperative care, Rare diseases, Spinal curvatures, Quality of life

## Abstract

**Background:**

Perioperative nursing care for patients with neuromuscular disorders, especially spinal muscular atrophy (SMA), remains a challenge. There is an obvious lack of guidelines.

**Methods:**

We retrospectively reviewed the medical charts of patients with type II or III SMA who underwent spinal surgery from 2018 to 2022. Nursing assessments included muscle strength, pulmonary function, the Barthel Index, the Braden Scale, Nutrition Risk Screening 2002, and the Hamilton Anxiety Scale. Preoperative and postoperative anxiety levels were compared using a paired-samples t-test.

**Results:**

All 24 included patients had severe scoliosis, kyphosis, or kyphoscoliosis, with a mean Cobb angle of 102 degrees. Upon admission, all patients (24/24) presented with muscle weakness, were classified as having total or severe dependency, and were at risk of developing pressure sores; 58.3% (14/24) of the patients had severe pulmonary function impairment, and 50.0% (12/24) were at nutritional risk, with the score unable to be assessed in 8.3% (2/24) of the patients. All patients underwent posterior spinal fusion surgery with bone grafting. Only one patient experienced a major postoperative complication, pneumonia, which was effectively managed. Anxiety level decreased significantly (*P* < 0.01) at discharge compared to that on admission. Complementing regular nursing care, an SMA-specific perioperative nursing care protocol was implemented: (1) Respiratory care protocol: A. Confirmation of SMA type; B. Comprehensive evaluation of symptoms, signs, and pulmonary function test results; C. Development and implementation of a personalized plan including: Plan 1. Training on respiratory function including diaphragmatic breathing exercise, coughing exercise, inhaling exercise, and exhaling exercise; Plan 2. Use of cough assist device, and/or Plan 3. Use of non-invasive ventilator. (2) Postoperative three-step all-involved training protocol of postural adaptation from nurse-led to caregiver-led and inducing patient self-advocacy: A. Preparation for the training; B. Postural adaptation training; C. Postural switch from lying to sitting.

**Conclusions:**

We implemented an SMA-specific perioperative nursing care protocol, including a respiratory care protocol and a postoperative three-step all-involved training protocol of postural adaptation, complementing standard nursing care. Our approach yielded positive patient outcomes, while we acknowledge the limitation that our protocol is pending comparative evaluations due to the rarity of the disease. The protocol was initially designed for patients with SMA but may also be suitable for other patients with profound muscle weakness.

**Supplementary Information:**

The online version contains supplementary material available at 10.1186/s13023-025-03718-z.

## Background

Spinal muscular atrophy (SMA) is a neurodegenerative autosomal recessive disorder characterized by progressive muscle atrophy. The reported incidence is approximately 1 in 10,000 live births [1]. SMA is mainly caused by pathogenic variants in the *SMN1* gene. A widely used clinical classification is based on the age of onset and maximum function achieved. Type II and Type III are less frequent than Type I, typically presenting at the ages of 7–18 months and 1.5–10 years, respectively [1]. Scoliosis, impaired respiratory function, and malnutrition are common findings in patients with SMA [2]. These conditions impose an additional disease burden and may be life-threatening.

Severe spinal deformities, such as  scoliosis, can lead to significant physical and psychological complications if untreated. Neuromuscular scoliosis, such as SMA-associated scoliosis, often presents as a more severe spinal deformity. These patients typically do not respond well to conservative treatments, leaving surgical intervention as the only effective option. However, increased incidences of perioperative complications have been observed in this population [3–5]. It has also been explicitly pointed out in the national consensus that even patients with milder SMA presentations may still experience respiratory infections during surgeries [6].

Nurses play a crucial role during the perioperative period. They work closely with patients and other healthcare professionals, provide comprehensive patient care, support the surgical team, and facilitate communication between patients, families or caregivers, and the healthcare team. A well-designed nursing protocol reduces complication rates and promotes optimal patient care outcomes. However, perioperative nursing care for patients with SMA remains challenging due to an obvious lack of guidelines.

In this study, we report our experience and a protocol for perioperative nursing care of patients with type II or type III SMA undergoing spinal surgery. All 24 patients enrolled in this study exhibited severe spinal curvature with muscle weakness and were classified as having either total or severe dependency. Significant impairment of pulmonary function was observed in 14 patients. Additionally, the majority of the patients were at risk for pressure ulcers and malnutrition. An SMA-specific perioperative nursing care protocol was implemented, complementing regular nursing care. This protocol includes a respiratory care protocol and a postoperative three-step all-involved training protocol of postural adaptation. Only one patient developed a major postoperative complication, pneumonia, which was largely attributed to her preexisting SMA-related comorbidities and was effectively managed. Anxiety level decreased significantly at discharge compared to that on admission. Our nursing care protocol yielded positive outcomes and may be suitable for other patients with significant muscle weakness, although we acknowledge the limitation that our protocol has not been comparatively evaluated due to the rarity of the disease.

## Methods

### Study design and ethics

This was a retrospective cohort study. We identified patients with a molecularly confirmed diagnosis of SMA receiving spinal surgery at Peking Union Medical College Hospital (PUMCH) from 2018 to 2022. Demographic characteristics and medical records were retrospectively reviewed. This study was approved by the Medical Ethical Committee of PUMCH (I-22PJ976), and written informed consent was obtained from the patients (18 years and older) or their legal guardians (under 18 years old).

### Nursing assessments

Muscle strength was assessed using the Medical Research Council Manual Muscle Testing scale, ranging from 0 to 5 [7]. Pulmonary function status was classified according to FEV1% pred, with FEV1 < 49% of predicted indicating severe impairment [8]. Patients who were physically unable to complete the pulmonary function test due to severe respiratory impairment were classified as having severe respiratory impairment. The level of dependency was assessed on admission using the Barthel Index (Supplementary Table 1) [9]. A score of 40 or less indicated severe impairment, a score between 41 and 60 indicated moderate impairment, a score between 61 and 99 indicated mild impairment, and a score of 100 indicated independency. Pressure sore risk was predicted using the Braden Scale on admission (Supplementary Table 2) [10]. According to the protocol at our center, a score of 12 or less was classified as a very high risk, a score between 13 and 18 was classified as a high risk, and a score higher than 18 was classified as a moderate risk. Nutrition risk screening was conducted utilizing Nutrition Risk Screening 2002 (NRS 2002) on admission (Supplementary Table 3) [11]. The cut-off score for the risk of malnutrition was equal to or greater than 3. Anxiety levels were measured by the Hamilton Anxiety Scale (HAMA) on admission and at discharge (Supplementary Table 4) [12]. A score higher than 29 might indicate severe anxiety; a score higher than 21 definitely indicated obvious anxiety; a score higher than 14 indicated anxiety; a score less than 6 indicated no anxiety. The cut-off point was 14.

### Statistical analysis

The anxiety levels before and after surgery were compared using a paired-samples t-test. A *p*-value of < 0.05 indicated statistical significance. Statistical analysis was performed using the stats package in R.

## Results

### Patient demographics

Between 2018 and 2022, 24 patients with type II or type III SMA received posterior spinal fusion for scoliosis at our center. Patient demographics are summarized in Table 1. Individual patient data is available in Supplementary Table 5. Of these patients, 14 were females and 10 were males, ranging in age from 10 to 28. There were 16 patients with SMA type II and 8 patients with SMA type III.

**Table 1:** Patient demographics and clinical characteristics

Characteristics	Number of patients (percentage)
Total	24 (100.0)
*Age (%), y/o*	
< 18	15 (62.5)
≥ 18	9 (37.5)
*Gender (%)*	
Female	14 (58.3)
Male	10 (41.7)
*Subtype (%)*	
II	16 (66.7)
III	8 (33.3)
*Cobb angle (%)*	
≥ 90°	15 (62.5)
< 90°	9 (37.5)
*Muscle Weakness (%)*	24 (100.0)
*Pulmonary function*^a^* (%)*	
Impairment	23 (95.8)
Severe impairment	14 (58.3)
Normal	1 (4.2)
*The Barthel Index (%)*	
Severe dependency	24 (100.0)
*The Braden Scale (%)*	
High risk	4 (16.7)
Moderate risk	20 (83.3)
*NRS 2002 (%)*	
At risk	12 (50.0)
Not at risk	10 (41.6)
Unable to assess^b^	2 (8.3)
*Preoperative HAMA (%)*	
Severe anxiety	1 (4.2)
Obvious anxiety	22 (91.6)
Anxiety	1 (4.2)
*Postoperative HAMA (%)*	
Severe anxiety	1 (4.2)
Obvious anxiety	3 (12.5)
Anxiety	20 (83.3)
*Without postoperative complications*	23 (95.8)

Data given as number of patients (percentage)

^a^Three patients were physically unable to complete the pulmonary function test and were therefore classified as having severe respiratory impairment

^b^NRS 2002 was unavailable in 2 patients since the body weight or height could not be measured due to the patients’ immobility or severe scoliosis

### Clinical characteristics

All patients (24/24) had severe scoliosis, kyphosis, or kyphoscoliosis, with a mean Cobb angle of 102 degrees. All patients (24/24) had prominent muscle weakness. For the pulmonary function test, three patients were physically unable to complete it due to severe respiratory impairment and were therefore classified as having severe respiratory impairment. Consequently, pulmonary function tests revealed that 95.8% of the patients (23/24) exhibited impaired pulmonary function, among which 58.3% of the patients (14/24) had severely impaired pulmonary function. Airway stenosis including tracheal deviation, recurrent airway infections including recurrent pneumonia, and/or asthma was observed in 37.5% of the patients (9/24). Overnight ventilatory support was required in 12.5% of the patients (3/24). All patients (24/24) were classified as total or severe dependency according to the Barthel Index, and all (24/24) were at risk of developing pressure sores as predicted by the Braden Scale. NRS 2002 scores were available for 22 patients, among whom 54.5% (12/22) were at nutritional risk. Nutritional states could not be assessed in two patients since they were not able to leave the bed due to severe spinal curvature or muscle weakness. All patients received posterior spinal fusion with bone grafting, and only one patient developed a major postoperative complication, pneumonia, which was effectively managed. Anxiety level decreased significantly (*P* = 5.983 × 10^−14^) at discharge compared to that on admission. Detailed clinical characteristics are available in Supplementary Table 5.

### SMA-specific personalized nursing care protocol

**Fig. 1:**
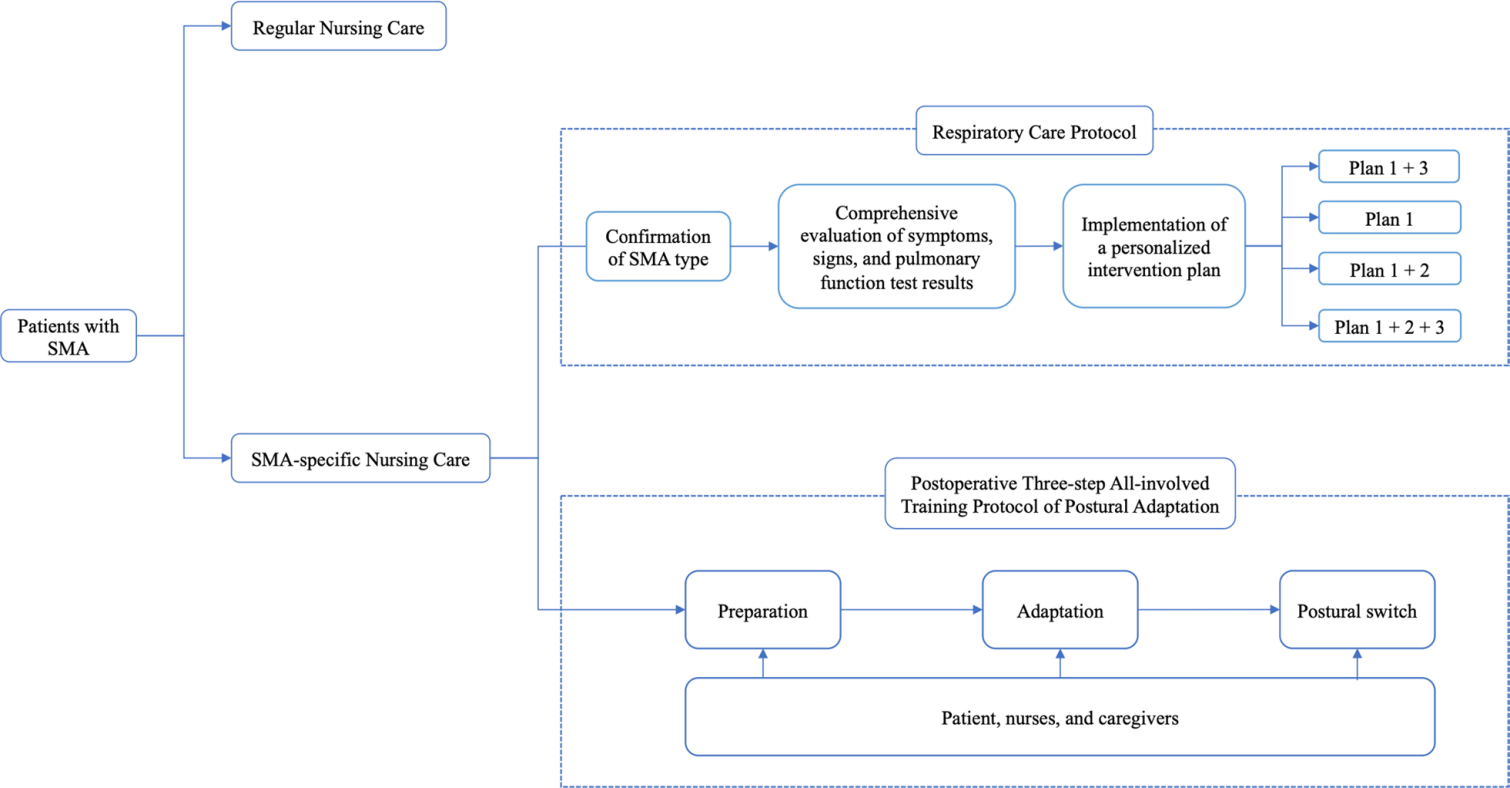
SMA-specific personalized Nursing Care Protocol

An SMA-specific personalized Nursing Care Protocol was developed considering the complexities of SMA. The protocol is summarized below, and an overview of the flowchart is shown in Fig. 1. The SMA-specific personalized Nursing Care Protocol is implemented complementing regular nursing care when a patient with SMA is admitted. The Protocol includes a respiratory care protocol and a postoperative three-step all-involved training protocol of postural adaptation.

1. Respiratory Care Protocol
2. A. Confirmation of SMA type

Pulmonary function is preliminarily evaluated based on the SMA subtype since SMA type III is usually a less severe form.

B. Comprehensive evaluation of symptoms, signs, and pulmonary function test results

In addition to the preoperative pulmonary function test, which is standard practice, special attention is given to a comprehensive evaluation of “symptoms and signs”. It includes the following assessments: recurrent pulmonary infections in the past three months, dyspnea, lung auscultation, sputum accumulation, cough effectiveness, characteristics of sputum, body temperature, and nocturnal hypoventilation. A detailed protocol is available in Supplementary Fig. 1.

      C. Implementation of a personalized intervention plan

Based on the results of the above-mentioned evaluation, a personalized intervention is implemented, including one of or a combination of the following three training plans.

Plan 1: Training on respiratory function.

**Fig. 2:**
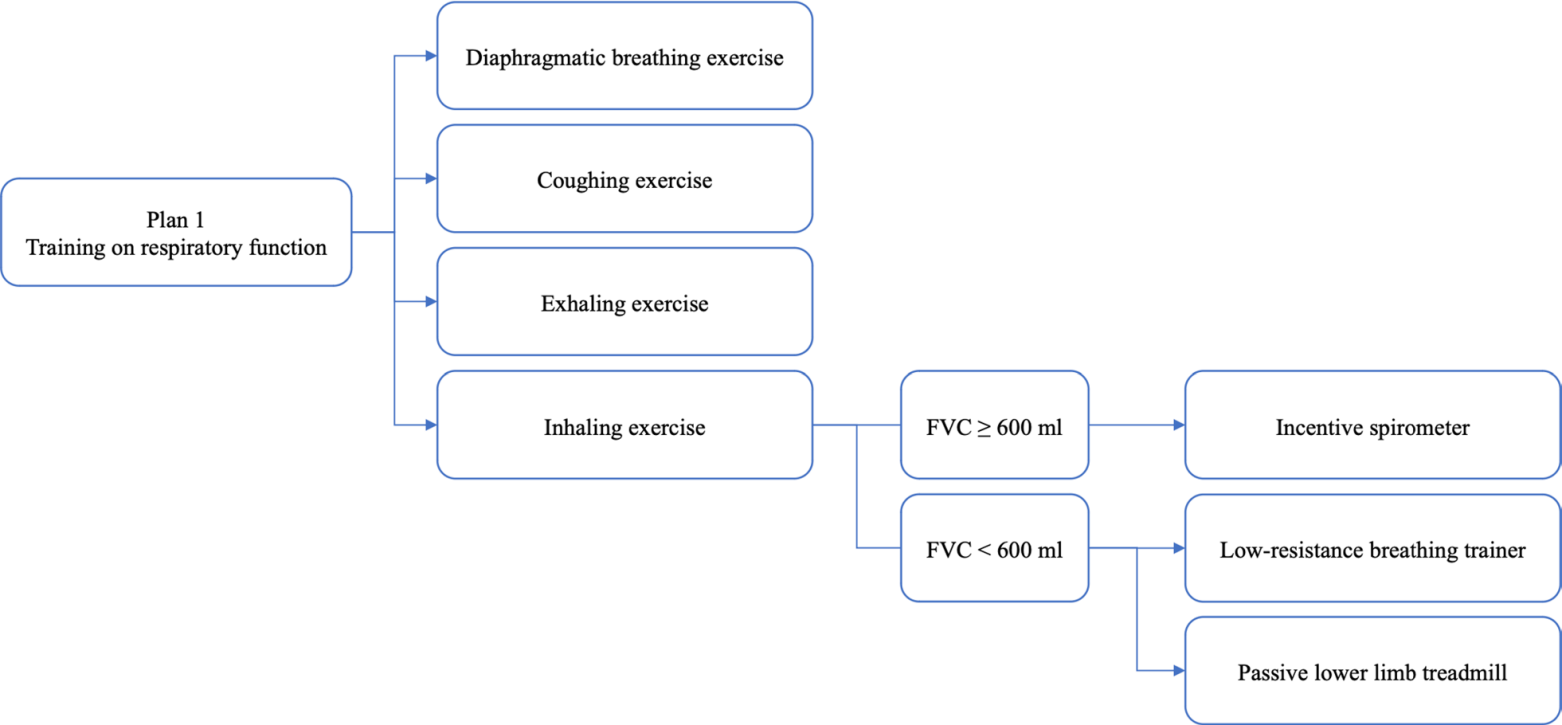
Training on respiratory function

The flowchart of training on respiratory function is shown in Fig. 2. During training, a “sitting position” is adopted to lower the position of the diaphragm and increase lung ventilation. This position helps increase the depth of breathing and the effectiveness of respiratory training. The patient is guided to do a diaphragmatic breathing exercise, which reduces the work of breathing muscles, increases the range of diaphragm movement, and improves ventilation function. The patient is taught to take a deep breath through the nose. Considering respiratory muscle weakness, the patient may be instructed to place his or her hands on the abdomen and inhale during the abdominal bulging to feel the change in strength. The inhalation to exhalation ratio is 1:3–4. Coughing exercise is carried out 10 times each in the morning, noon, and evening. It comprises taking a deep breath, holding it, and then forcefully coughing 2–3 times using the abdominal muscles. Exhalation exercise involves blowing paper through a pursed lip, 10 sets each time, 2–3 times a day. For inhaling exercise, a patient with an FVC equal to or greater than 600 ml is instructed to use incentive spirometers. For a patient with an FVC of less than 600 ml and weak respiratory muscle strength, a “low-resistance breathing trainer” and a passive lower limb treadmill are recommended. When training with devices like POWERbreathe®, the resistance is adjustable. Using an appropriate training load, the patient is instructed to place the mouthpiece in the mouth, and then take a fast, forceful breath in to expand the chest. For exhalation, the patient should breathe out slowly and passively through the mouth to empty the lungs, then pause for about 3–4 s until feeling the urge to inhale again. This training is performed twice a day for 30 times each set, and a training diary is kept to improve patient compliance. If the patient experiences wheezing, dizziness, or coughing during training, he or she is instructed to take a short break. This training process is tolerable for most patients. A passive lower limb treadmill trains the diaphragm to improve respiratory muscle strength and endurance, achieving the goal of inspiratory muscle training. It is performed using lower limb exercise equipment with four movements including “leg pedaling, leg splitting, hip flexion, and leg extension”, 10 times per set, and a total of 3 sets every day. Nurses instruct and evaluate the patient’s tolerance at the bedside throughout the entire training.

Plan 2: Use of cough assist device.

It is widely acknowledged that patients with type II and type III SMA having difficulty with independent sputum clearance should be evaluated for the use of a cough assist device. Insufflation and exsufflation pressures are adjusted based on the patient’s pulmonary function and sputum clearance ability. The nurse first evaluates the patient’s and caregiver’s understanding and usage of the cough assist device. Then the nurse explains the two phases of the device: during phase one, positive air pressure is applied to expand the lungs and loosen the bronchial secretions; during phase two, negative pressure pushes secretions toward the large airways for clearance by suction. The pressure is typically set at ± 25–40 cmH_2_O, depending on the subtype and the patient's tolerance. At the same time, the patient and caregiver are instructed to pay attention to: positioning the patient in a semi-recumbent or upright position, stabilizing the neck to avoid cervical instability during suction; clearing oral and nasal secretions prior to using the device, either before meals or at least one hour after meals; performing at least 10 cough cycles in each session, and at least two sessions each day. The patient and caregiver are encouraged to participate together and practice multiple times. A flowchart for utilizing the cough assist device is shown in Supplementary Fig. 2.

Plan 3: Use of non-invasive ventilator.

Non-invasive ventilation treatment is used during nighttime sleep for SMA patients with nocturnal hypoventilation. This includes snoring, waking up due to breathing difficulties during sleep, feeling sleepy and fatigued upon waking up in the morning, and mild carbon dioxide retention as detected by blood gas analysis. A flowchart for utilizing the non-invasive ventilation is shown in Supplementary Fig. 3.

2. Postoperative three-step all-involved training protocol of postural adaptation

**Fig. 3:**
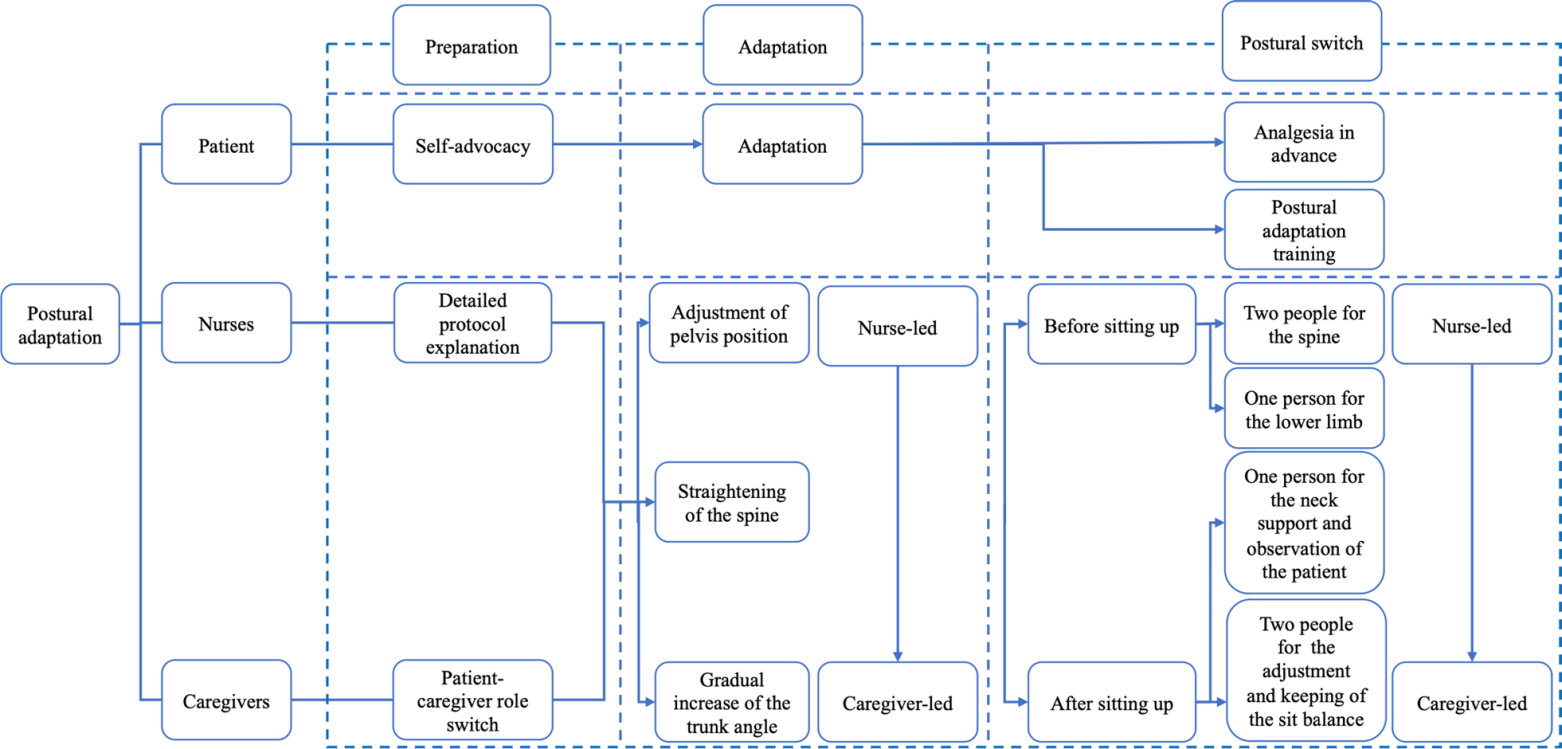
Postoperative three-step all-involved training protocol of postural adaptation

The postural adaptation protocol includes three steps and requires participation from the patient, caregiver, and nurses, as shown in Fig. 3 and Supplementary Figs. 4 and 5.

A. Preparation for the training

The patient should take an active role during the entire process. The nurses explain the detailed protocol to the patient and caregiver, helping them gain a better understanding of the protocol and what to expect from the training. The caregiver is instructed to do a patient-caregiver role switch to appreciate the patient’s experiences and challenges.

B. Postural adaptation training.

In this section, the patient gradually adapts to sitting balance. The training shifts from nurse-led to caregiver-led in a gradual and collaborative manner. The essential elements involve optimizing pelvic alignment, maintaining spinal alignment, progressively increasing head-of-bed elevation and duration while adjusting the plan based on the patient’s tolerance (Supplementary Fig. 4). This approach enhances the patient’s autonomic nervous system modulation and strengthens the paraspinal muscles, facilitating the gradual adjustment to sitting balance.

C. Postural switch from lying to sitting.

This section involves completing the position change with a focus on “safety + comfort” (Supplementary Fig. 5). Before the position change, the patient receives appropriate analgesia. Three nurses work together to complete the postural switch: the nurse near the head of the bed is responsible for the shoulders and waist, the nurse near the foot of the bed is responsible for the lower limbs, and the nurse on the opposite side is responsible for overall assistance and safety. Given the weak head control ability of patients with SMA, the nurse on the opposite side must also support and protect the neck of the patient after sitting to enhance stability and balance. Attention should be paid to any discomfort observed during the position change. Sitting time is gradually increased according to the patient’s tolerance. The training also shifts from nurse-led to caregiver-led. The nurses assess the caregiver’s learning outcomes to enhance their proficiency in long-term home care.

## Discussion

Providing perioperative nursing care for spinal surgery in SMA patients with severe spinal deformities and preexisting comorbidities has been a longstanding challenge. This study reported our experience in the perioperative nursing care of patients with type II or type III SMA undergoing spinal surgery at our center. An SMA-specific perioperative nursing care protocol was implemented to complement standard nursing care. The protocol includes a respiratory care protocol and a postoperative three-step all-involved training protocol of postural adaptation. All 24 patients who received surgeries presented with severe spinal curvature and a variety of comorbidities associated with SMA. Only one patient experienced a major postoperative complication, which was pneumonia. It was largely related to known preoperative conditions and was effectively and successfully managed. In addition, there was a significant decrease in anxiety levels observed at discharge compared to admission. The patients finally achieved an upright sitting posture, which is always an important objective for SMA patients and their families. We observed satisfactory outcomes with our perioperative nursing care, although we acknowledge that further comparative evaluation would be beneficial for obtaining a more comprehensive understanding of its impact.

The prevalence of spinal deformities is high in patients with SMA. The lifetime probability of undergoing spinal surgery, such as scoliosis surgery, varies among subtypes, with the highest likelihood observed in Type II SMA, exceeding 80% [13]. Newly developed treatment options, such as Nusinersen, significantly modify the natural course of SMA. It has been reported that patients with type I who receive early treatment may achieve independent sitting but are also at an increased risk of developing early-onset spinal deformities [14]. During clinical evaluations, SMA patients often present with severe spinal deformities and may need surgical interventions.

Statistical data regarding postoperative complications in patients with SMA is limited, largely due to the rarity of the disease. Studies on patients with neuromuscular scoliosis, including scoliosis secondary to SMA, have revealed an overall increased risk of postoperative complications. A study based on the Scoliosis Research Society (SRS) Morbidity and Mortality database reported a 3.5-fold decrease in complication rate, from approximately 15% to 4%, between 2004–2007 and 2012–2015 [15]. However, the authors acknowledged a lower incidence of complications compared to other studies possibly due to underreporting [15]. The complication rates reported in recent cohort studies vary from 27 to 43% [3–5]. Well-documented risk factors associated with higher risks of postoperative complications include nonambulatory status, preoperative curve magnitude, malnutrition, and preexisting pulmonary compromise [3, 16–18]. These factors are commonly found in patients with SMA, presenting even greater difficulties to orthopedic teams.

Among the reported complications, pulmonary issues are among the most prevalent, accounting for 5 to 14% of the complications [3, 4]. These include prolonged atelectasis, prolonged intubation, pneumonia or bronchopneumonia, and respiratory failure. A study on neuromuscular scoliosis surgery, including five patients with pulmonary comorbidities, reported complications in three out of these five patients [3]. Severe pulmonary comorbidities are common in patients with SMA, and approximately 20–45% of patients with type II SMA and 5% patients with type III SMA requiring ventilatory support [2, 19, 20]. A retrospective study reviewed the medical records of 16 children with SMA who underwent spinal surgeries, with preoperative major cobb angles of 78 ± 20 degrees [21]. The study identified one case of reintubation for low tidal volumes and one case of postoperative pneumonia. In another cohort study involving 22 patients with type II SMA presenting with a mean preoperative Cobb angle of 59.3 degrees, the reported postoperative complications included seven cases of atelectasis and one case of pneumonia [22]. In our cohort of 24 patients with a mean Cobb angle of 102 degrees, only one patient experienced a major postoperative complication, which was pneumonia (Supplemental Table 5). This patient had a typical and severe presentation of type II SMA. Before the surgery, she had upper respiratory tract infection, severely impaired pulmonary function and was on nocturnal ventilatory support. She was nutritionally at risk and bed-bound due to extreme muscle weakness and severe spinal deformities. The postoperative pneumonia was largely associated with her preexisting SMA-related comorbidities and was effectively managed by our team.

Nurses make substantial contributions to the orthopedic team. Their specialized, continuous, and accountable care supports patients and caregivers clinically and emotionally [23]. Considering the pre-existing pulmonary conditions, accurate evaluation of preoperative lung function is crucial for SMA patients undergoing spinal surgeries. Currently, the assessment largely relies on pulmonary function tests. It requires the patient’s ability to perform the test and may not be sufficient for fully assessing SMA patient’s respiratory function due to the significantly impaired lung function. It has been recommended by national consensus to integrate clinical indicators such as SpO_2_ into comprehensive assessments of pulmonary function for patients with SMA [24]. However, no specific approach has been recommended to achieve this comprehensive assessment. Our respiratory care protocol emphasizes the importance of a comprehensive evaluation of “symptoms and signs”. Features such as the frequency and severity of pulmonary infections, lung auscultation, and cough effectiveness may provide additional information, particularly in patients who are unable to complete the pulmonary function test. Taking all these factors into consideration gives a more comprehensive picture of the patient’s actual lung function. The personalized training on respiratory function is then designed based on the evaluation results. Practicing rhythmic breathing is critical and essential. Patients with SMA may present with a high respiratory rate and unsynchronized breathing due to respiratory muscle weakness. Restrictive ventilatory dysfunction is a common finding. These factors lead to difficulties in taking a deep breath, resulting in an “inefficient breathing pattern”. The training helps patients re-establish a rhythmic breathing pattern, effectively enhances lung function in a short term, improves tolerance of spinal surgery, and reduces the incidence of postoperative pulmonary complications.

Patient care comprises not only nursing assessment, but also effective care coordination and patient education [23]. In recent years, there has been a growing emphasis on prioritizing wellness and prevention in healthcare. An effective nursing care protocol not only focuses on the patient’s current presentation, but also goes further and addresses “up-stream” factors, thereby improving patient outcomes [25]. Shifting towards patient-centered and coordinated health care has been a hot topic recently. Nurses play a critical role by developing and implementing effective nursing care protocols. Holistic care strengthens the patient’s capacity and empowers the patient to manage the disease after discharge, thereby achieving life goals. It is often difficult for patients with SMA to maintain a sitting position before the surgery due to progressive muscle weakness and atrophy, as well as large Cobb angles and pelvic imbalances. Only a few patients can sit independently for 20–30 seconds with the assistance of caregivers or braces. The surgery corrects or reduces the spinal curves. Patients with other types of (kypho)scoliosis without systemic involvements, such as adolescent idiopathic scoliosis, typically achieve postoperative sitting and standing within two to three days without any assistance. However, for patients with SMA, the SMA-associated muscle weakness and atrophy, reduced proprioception resulting from prolonged bed rest, together with significant pain and other injuries due to the invasive nature of the surgery post challenges to achieving sitting balance. Therefore, a well-designed protocol is essential. Patient self-advocacy is an important factor in the whole process of postural adaptation, as active engagement can lead to better outcomes. The postoperative three-step all-involved training protocol of postural adaptation in our protocol takes into account the patient’s goals and limitations and emphasizes the involvement of the patient, caregiver, and nurses, thereby effectively helping the patients to achieve sitting balance. The patient is prepared for the training both physically and psychologically, with appropriate analgesia administered to reduce pain and the self-motivation to complete the postural switch. At the same time, caregivers are educated to effectively communicate with the patient and understand the patient’s unique needs through the patient-caregiver role-reversal exercise. And then the training shifts from being nurse-led to caregiver-led. Besides, at our center, caregivers are typically family members. The hospital does not charge an additional fee for the assistance, which alleviates concerns about insurance coverage or socio-economic burden. At our center, 30% of patients who were able to sit independently before the surgery adapted to postural balance after orthotic correction; 60% of patients who required assistance to sit before the surgery regained postural balance after orthotic correction; and 10% of patients who were unable to sit independently before the surgery gained assisted postural balance. Further, chronically ill patients, including patients with SMA, often require continuous and long-term care. Instructed by experienced nurses, caregivers are equipped with professional care skills necessary to ensure patient safety, enhance quality of life, and enable both the patients and the caregivers to live to the fullest potential.

The protocol was initially designed for patients with SMA but may also be suitable for other patients with profound muscle weakness. Spinal deformities requiring surgical interventions are also frequently observed in patients with other neuromuscular diseases, such as Duchenne muscular dystrophy, Congenital Muscular Dystrophy, Charcot-Marie-Tooth disease, and in senior patients [26–29]. These patients are also likely to have preexisting conditions such as loss of ambulation, respiratory deterioration, and decreased body weight, which are identified as risk factors for perioperative complications [18, 30]. The fundamental principle behind our protocol for patients with SMA is similar to that for patients with the above-mentioned conditions. Therefore, the current protocol can be slightly modified and applied to patients with these various conditions, with the anticipation of recuding perioperative complications and improving quality of life.

## Limitations

The conclusions drawn from this study should be interpreted with caution, as there were several limitations in the method, largely due to the rarity of SMA. The primary limitation stems from the small sample size, which may lead to cognitive bias. With only 24 identified patients, subgroup comparisons based on assessment results such as muscle strength and pulmonary function were unfeasible. Case–control studies may provide stronger evidence, but we were not able to identify other patients before the implementation of our nursing protocol. Besides, there is very limited evidence reporting perioperative complications or mortality rates in patients with SMA undergoing spinal surgeries, particularly in China. And it is well known that the surgical and nursing care plans vary significantly between countries, and considerably influence perioperative complications and mortality rates. Therefore, at this stage, we are unable to conduct a meaningful comparative evaluation between the data from our cohort and that from the limited available literature to demonstrate a statistically significant reduction in complication rates and anxiety levels. Additionally, although the anxiety level decreased significantly at discharge, we can not rule out the possibility that the patients were more anxious on admission because of the fear about the upcoming surgery.

Nevertheless, our nursing protocol improves outcomes in patients with SMA, and may benefit other patients with profound muscle weakness.

## Conclusion

Complementing standard nursing care, we implemented an SMA-specific perioperative nursing care protocol, including a respiratory care protocol and a postoperative three-step all-involved training protocol of postural adaptation. All 24 patients receiving surgeries presented with severe spinal curvature and various comorbidities associated with SMA. Only one patient experienced a major postoperative complication primarily related to pre-existing conditions and was effectively managed. In addition, postoperative anxiety levels decreased significantly compared to that on admission. The patients finally achieved an upright sitting posture, achieving the important goal of patients and their families. Our perioperative nursing care yielded satisfactory outcomes. The protocol was initially designed for patients with SMA but may also be suitable for other patients with profound muscle weakness. Further comparative evaluation would deepen our understanding of the protocol’s impact on patients with SMA, as well as on individuals with other conditions leading to muscle weakness.

## Supplementary Information

Additional file 1.

Additional file 2.

Additional file 3.

Additional file 4.

Additional file 5.

Additional file 6.

Additional file 7.

Additional file 8.

Additional file 9.

Additional file 10.

## Data Availability

The datasets supporting the conclusions of this article are included within the article and its additional files.

## Abbreviations

SMA: Spinal muscular atrophy
HAMA: Hamilton Anxiety Scale
SRS: Scoliosis Research Society
