# Supplementary figures and images for: A perioperative nursing care protocol for patients with spinal muscular atrophy (SMA) type II or type III undergoing spinal surgery: a 4-year experience in 24 patients
Source: Orphanet J Rare Dis. 2025 May 19;20:237. doi: 10.1186/s13023-025-03718-z (PMC12087051; doi:10.1186/s13023-025-03718-z)

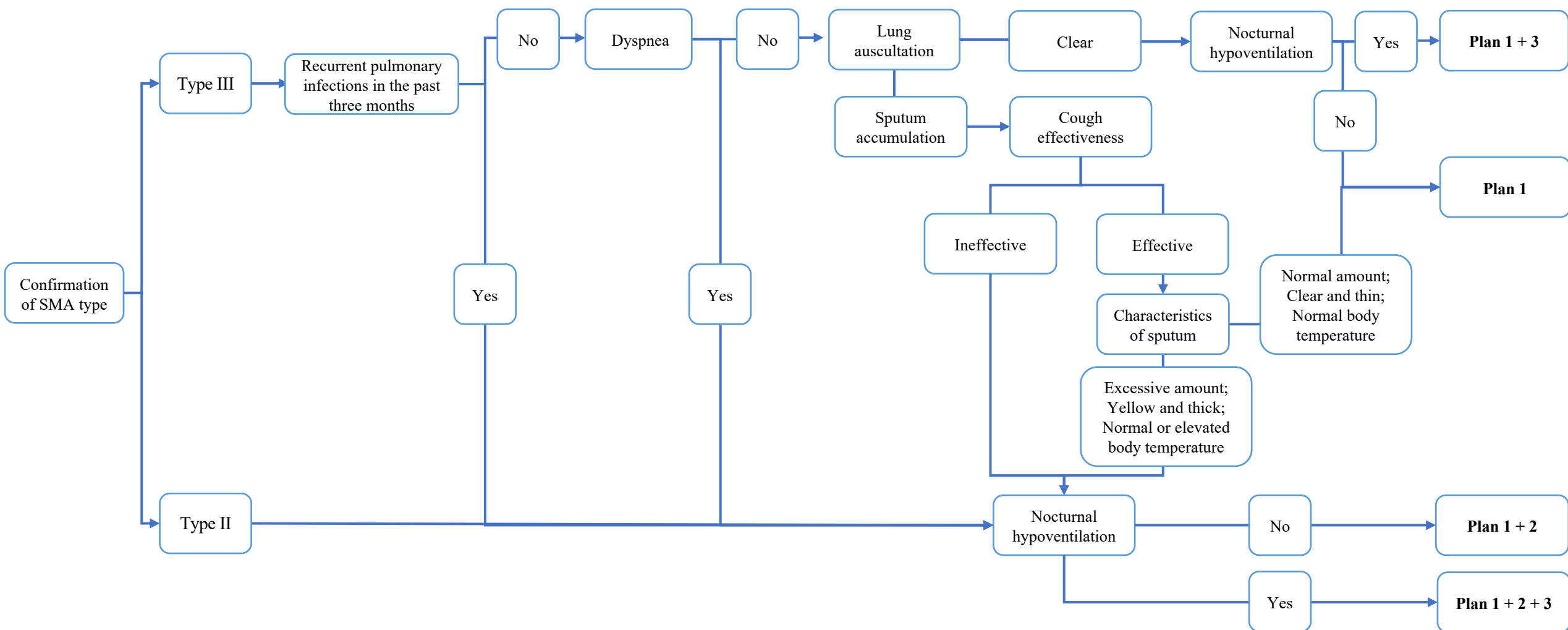

**Supplementary Figure 1. Detailed respiratory care protocol**

Supplement: Supplementary file 1 — Additional file 1. [file 13023_2025_3718_MOESM1_ESM.pdf]

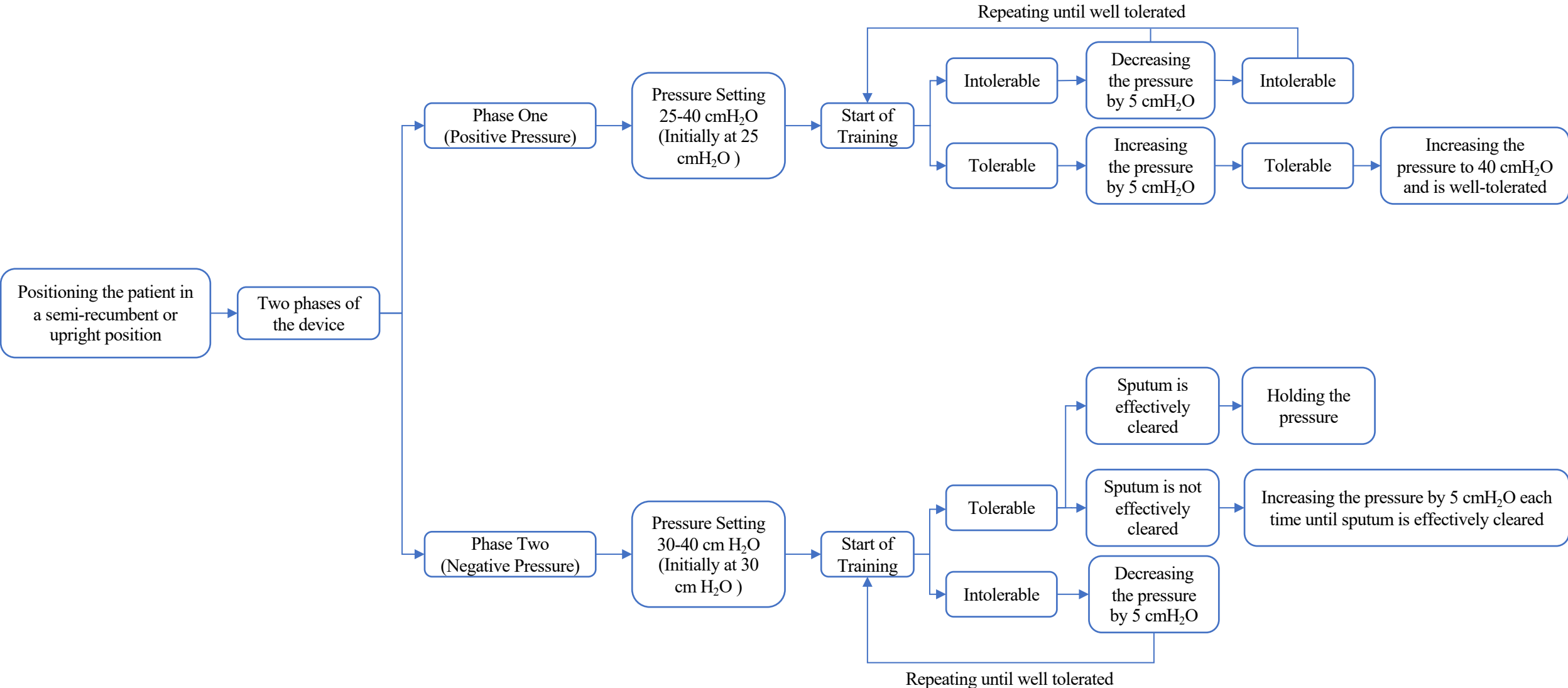

**Supplementary Figure 2. Flowchart for utilizing the cough assist device**

Supplement: Supplementary file 2 — Additional file 2. [file 13023_2025_3718_MOESM2_ESM.pdf]

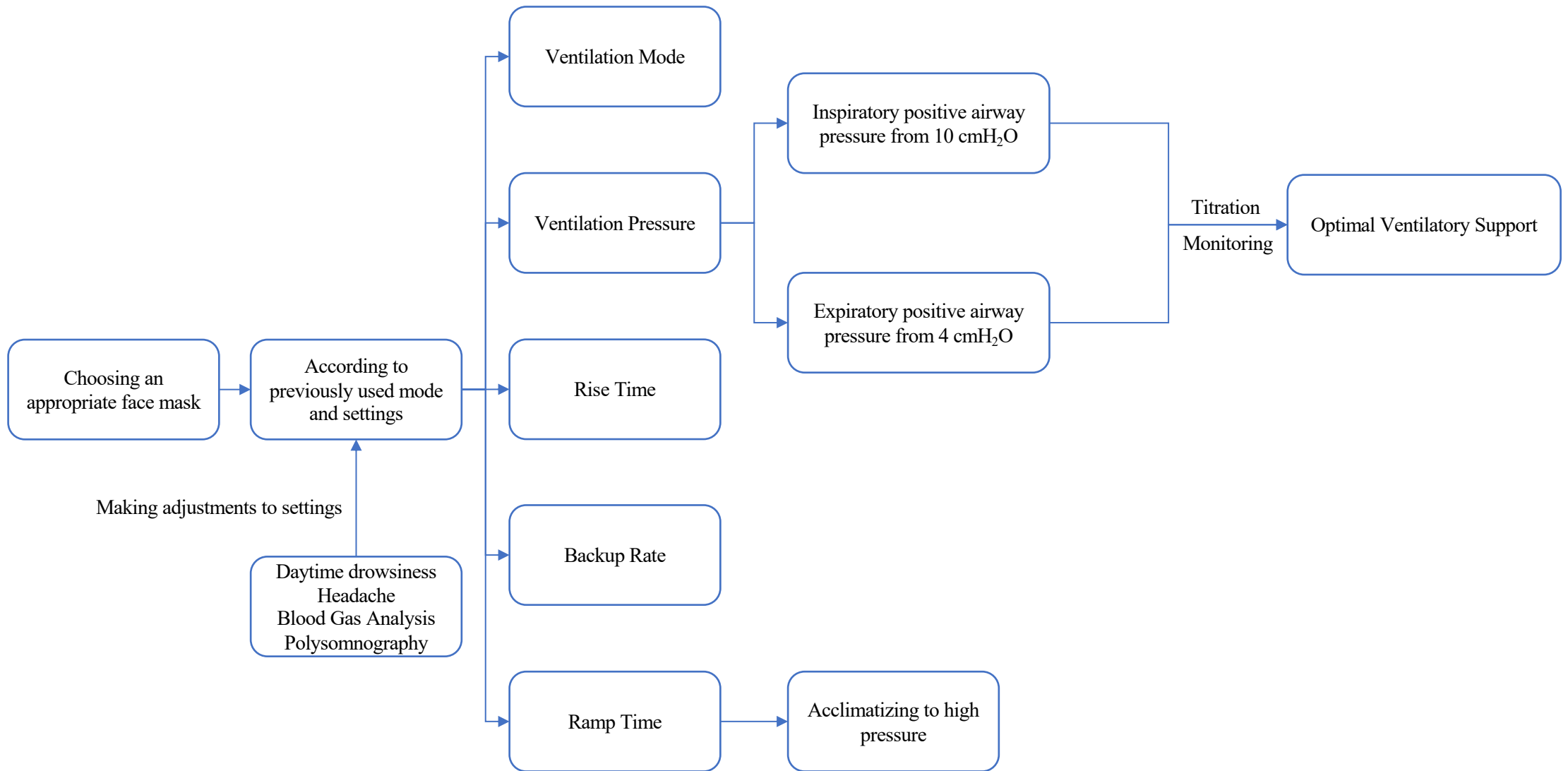

**Supplementary Figure 3. Flowchart for utilizing the non-invasive ventilation**

Supplement: Supplementary file 3 — Additional file 3. [file 13023_2025_3718_MOESM3_ESM.pdf]

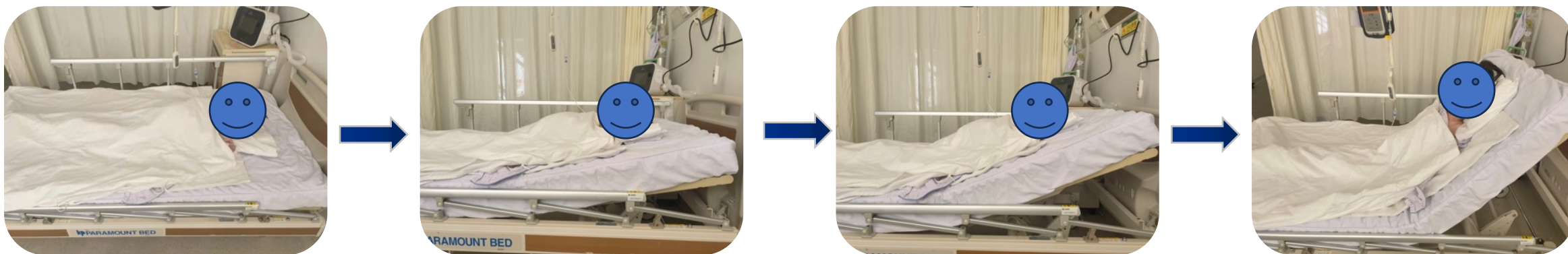

**Supplementary Figure 4. Postural adaptation training**

Supplement: Supplementary file 4 — Additional file 4. [file 13023_2025_3718_MOESM4_ESM.pdf]

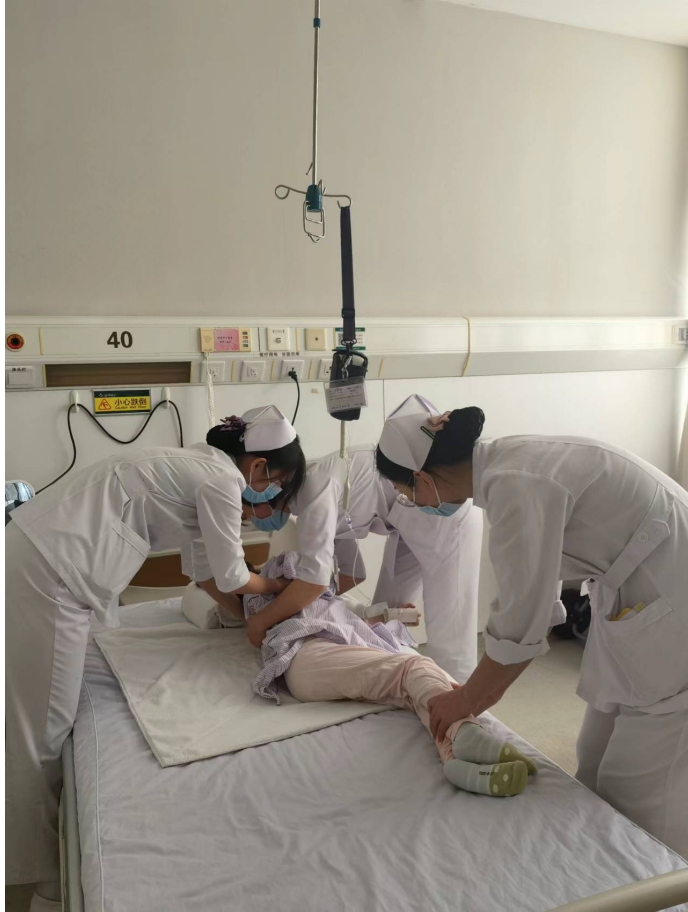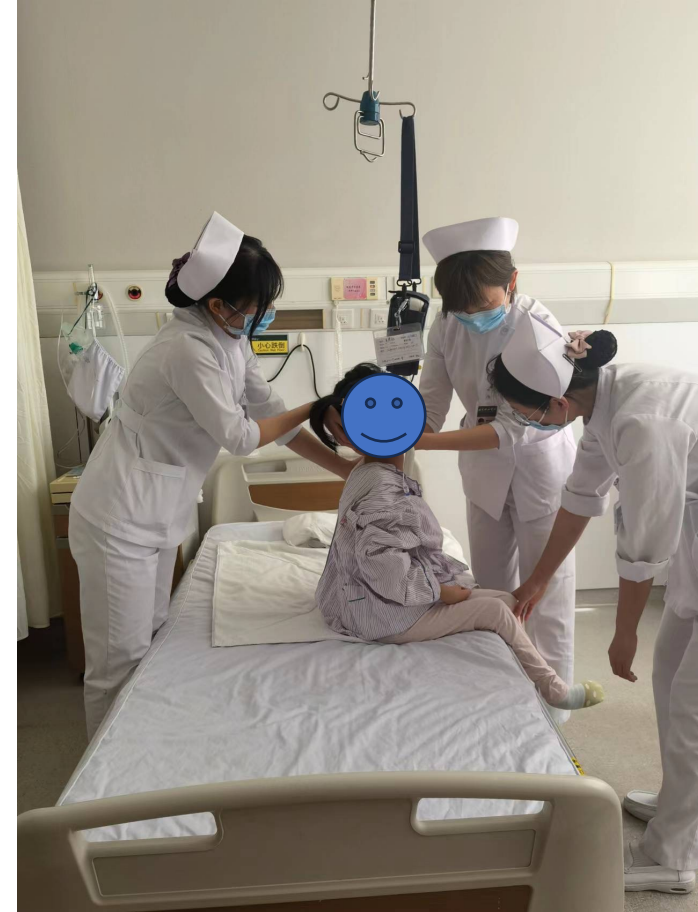

**Supplementary Figure 5. Postural switch from lying to sitting**

Supplement: Supplementary file 5 — Additional file 5. [file 13023_2025_3718_MOESM5_ESM.pdf]
