# Supplementary material for: A perioperative nursing care protocol for patients with spinal muscular atrophy (SMA) type II or type III undergoing spinal surgery: a 4-year experience in 24 patients
Source: Orphanet J Rare Dis. 2025 May 19;20:237. doi: 10.1186/s13023-025-03718-z (PMC12087051; doi:10.1186/s13023-025-03718-z)
Supplement: Supplementary file 6 — Additional file 6. [file 13023_2025_3718_MOESM6_ESM.docx]

Supplementary Table 1. The Barthel Index

| Department________ | | Patient Name________ | | Gender________ | |
| --- | --- | --- | --- | --- | --- |
| Bed No.______ | | Medical Record ID No.______ | | Assessment Date______ | |
| Time Point: | □ On Admission | □ After Transfer | □ Pre-operation | □ Post-operation | □ At Discharge |

| Items | Check the appropriate box corresponding to the patient’s actual level of ability | | | |
| --- | --- | --- | --- | --- |
| 1. Feeding | □Independent (10) | □ Minor help (5) | □ Dependent (0) |  |
| 2. Bathing | □Independent (5) | □ Dependent (0) |  |  |
| 3. Grooming | □Independent (5) | □ Dependent (0) |  |  |
| 4. Dressing | □Independent (10) | □ Minor help (5) | □ Dependent (0) |  |
| 5. Controlling bowels | □Independent (10) | □ Minor help (5) | □ Major help (0) |  |
| 6. Controlling bladder | □Independent (10) | □ Minor help (5) | □ Major help (0) |  |
| 7. Toilet Use | □Independent (10) | □ Minor help (5) | □ Major help (0) |  |
| 8. Transfers between bed and chair | □Independent (15) | □ Minor help (10) | □ Major help (5) | □ Dependent (0) |
| 9. Mobility on level surfaces | □Independent (15) | □ Minor help (10) | □ Major help (5) | □ Dependent (0) |
| 10. Ascending and descending stairs | □Independent (10) | □ Minor help (5) | □ Major help (0) |  |

| Total Score: ________ |  |
| --- | --- |
| Degree: ________ | Signature of Nurse: ________ |

| Criteria for Degree Classification |  |  |
| --- | --- | --- |
|  | 1. Total Score: | Sum the score for each item |
|  | 2. Degree: | 0 = Independency: 100, able to perform activities of daily living without assistance |
|  |  | 1 = Mild Impairment: 61–99, able to perform some activities of daily living independently with some assistance |
|  |  | 2 = Moderate Impairment: 41–60, unable to perform activities of daily living without major assistance |
|  |  | 3 = Severe Impairment: ≤ 40, unable to perform major activities of daily living or total dependent |
