# Supplementary material for: A perioperative nursing care protocol for patients with spinal muscular atrophy (SMA) type II or type III undergoing spinal surgery: a 4-year experience in 24 patients
Source: Orphanet J Rare Dis. 2025 May 19;20:237. doi: 10.1186/s13023-025-03718-z (PMC12087051; doi:10.1186/s13023-025-03718-z)
Supplement: Supplementary file 7 — Additional file 7. [file 13023_2025_3718_MOESM7_ESM.docx]

Supplementary Table 2. The Braden Scale

| Department________ | | Patient Name________ | | Gender________ | |
| --- | --- | --- | --- | --- | --- |
| Bed No.______ | | Medical Record ID No.______ | | Assessment Date______ | |
| Time Point: | □ On Admission | □ After Transfer | □ Pre-operation | □ Post-operation | □ At Discharge |

| Items | Score and Description | | | | Please Indicate Appropriate Numbers |
| --- | --- | --- | --- | --- | --- |
|  | 1 | 2 | 3 | 4 |  |
| Sensory Perception | Completely Limited | Very Limited | Slightly Limited | No Impairment |  |
| Degree of Moisture | Constantly Moist | Often Moist | Occasionally Moist | Rarely Moist |  |
| Activity | Bedfast | Chairfast | Walks Occasionally | Walks Frequently |  |
| Mobility | Completely Immobile | Very Limited | Slightly Limited | No Limitation |  |
| Nutritional Intake | Very Poor | Probably Inadequate | Adequate | Excellent |  |
| Friction and Shear | Problem | Potential Problem | No Apparent Problem |  |  |
| Total Score: ________ | | | | | |

| Preventive Measures | Educate the patient and their family members about the potential risks of developing pressure ulcers and provide instructions regarding preventive measures. | □ |
| --- | --- | --- |
|  | Implement regular turning and repositioning, reduce pressure on the skin, and avoid friction. | □ |
|  | Utilize tools such as (1) air cushions, (2) polymer pads, (3) soft pillows, and (4) protective films. | ( ) |
|  | Keep skin and bed linen clean and dry. | □ |
|  | Provide guidance and assistance to patients during repositioning to avoid skin tension and friction. | □ |
|  | Instruct patients and their families on appropriate dietary choices and enhancement of nutrition. | □ |
| Preventive Outcomes | Intact skin | □ |
|  | Skin exhibiting erythema that resolves upon pressure relief | □ |
|  | Localized skin presents with erythema, swelling, induration, or purplish ecchymosis. |  |
|  | Presence of blistering or skin breakdown |  |

| Signature of Nurse: ________ |
| --- |
