# Supplementary material for: A perioperative nursing care protocol for patients with spinal muscular atrophy (SMA) type II or type III undergoing spinal surgery: a 4-year experience in 24 patients
Source: Orphanet J Rare Dis. 2025 May 19;20:237. doi: 10.1186/s13023-025-03718-z (PMC12087051; doi:10.1186/s13023-025-03718-z)
Supplement: Supplementary file 8 — Additional file 8. [file 13023_2025_3718_MOESM8_ESM.docx]

Supplementary Table 3. Nutritional Risk Screening 2002

| Disease Effect Score  Score: ________ | 1 Point | □ Hip fracture | □ Acute exacerbation or complication of chronic disease | | | | | | □ COPD |
| --- | --- | --- | --- | --- | --- | --- | --- | --- | --- |
|  |  | □ Dialysis | □ Cirrhosis | | □Malignant tumor | | | | □ Diabetes |
|  |  | □ A patient with chronic disease admitted to hospital due to complications. The patient is weak but out of bed regularly. Protein requirement is increased but can be supplemented by oral diet. | | | | | | | |
|  | 2 Points | □ Major abdominal surgery | | □ Stroke | | □ Severe pneumonia | | □ Hematologic malignancy | |
|  |  | □ A patient confined to bed due to illness, e.g., following major abdominal surgery. Protein requirement is substantially increased but can be covered through parenteral nutrition or eternal nutrition. | | | | | | | |
|  | 3 Points | □ Brain Injury | □ Bone marrow transplantation | | | | □ Intensive care patients with APACHE > 10 | | |
|  |  | □ A patient in intensive care with assisted ventilation. Protein requirement is increased and cannot be supplemented by parenteral nutrition or eternal nutrition. Protein breakdown and nitrogen loss can be significantly attenuated. | | | | | | | |
|  | 0 Point | □ None of the above | | | | | | | |

| Nutritional Status  Score: ________ | 1. | ① BMI (kg/m^2^) | □ < 18.5 (3 Points) | | | |
| --- | --- | --- | --- | --- | --- | --- |
|  |  | □ Note: When the accurate BMI value cannot be obtained due to severe pleural effusion, ascites, or edema, and there is no severe impairment of liver or renal function, albumin can be used instead. ____(g/L) (< 30 g/L, 3 Points) | | | | |
|  |  | ② Weight loss > 5% within: | | □ 3 months (1 Point) | □ 2 months (2 Point) | □ 1 months (3 Point) |
|  |  | ③ Food intake decreased in the last week: | | □ 25%–50% (1 Point) | □ 51%–75% (2 Point) | □ 76%–100% (3 Point) |
|  | 2. | 0 Point | □ None of the above | | | |

| Age Score: ________ | □ ≥ 70 years (1 Point) | □ < 70 years (0 Point) |
| --- | --- | --- |

Total Score: ________

For diseases that are not clearly listed in the table, refer to the following criteria and scores according to the investigator's interpretation.

1Point: A patient with chronic disease admitted to hospital due to complications. The patient is weak but out of bed regularly. Protein requirement is increased but can be supplemented by oral diet.

2 Points: A patient confined to bed due to illness, e.g., following major abdominal surgery. Protein requirement is substantially increased but can be covered through parenteral nutrition or eternal nutrition in most of the cases.

3 Points: A patient in intensive care with assisted ventilation. Protein requirement is increased and cannot be supplemented by parenteral nutrition or eternal nutrition. But protein breakdown and nitrogen loss can be significantly attenuated with parenteral nutrition or eternal nutrition.

When the total score ≥ 3 points: The patient is at nutritional risk and requires nutritional support. Nurses should formulate nutritional support plans in combination with clinical practice.

When the total score < 3 points: Review nutritional risk screening weekly.
