# Supplementary material for: A perioperative nursing care protocol for patients with spinal muscular atrophy (SMA) type II or type III undergoing spinal surgery: a 4-year experience in 24 patients
Source: Orphanet J Rare Dis. 2025 May 19;20:237. doi: 10.1186/s13023-025-03718-z (PMC12087051; doi:10.1186/s13023-025-03718-z)
Supplement: Supplementary file 9 — Additional file 9. [file 13023_2025_3718_MOESM9_ESM.docx]

Supplementary Table 4. Hamilton Anxiety Scale (HAMA)

Hamilton Anxiety Scale (HAMA) was developed by Hamilton in 1959.(1) It is one of the commonly used scales in clinical psychiatry, including 14 items.

[Items and Evaluation Standards] All HAMA items use a 5-level scoring method from 0 to 4 points. The standards for each level are:

| (0) Not Present; | (1) Mild; | (2) Moderate; | (3) Severe; | (4) Very Severe |
| --- | --- | --- | --- | --- |

| 1. Anxious Mood | Worries, anticipation of the worst, irritability. |
| --- | --- |
| 2. Tension | Feelings of tension, fatigability, unable to relax, startle response, moved to tears easily, trembling, feeling anxious. |
| 3. Fear | Fearful of the dark, strangers, being alone, animals, traffic or travel, and crowds. |
| 4. Insomnia | Difficulty falling asleep, sleep disturbance, lack of deep sleep, excessive dreaming, nightmare, night terror, fatigue on waking. |
| 5. Cognitive Function | Also known as memory, attention disturbance. Difficulty in concentration, poor memory. |
| 6. Depressed Mood | Loss of interest, lack of pleasure in past hobbies, depression, early waking, diurnal variation. |
| 7. Muscular System Symptoms | Muscle soreness, body inflexibility, muscle twitching, limb twitching, myoclonic jerks, grinding of teeth, unsteady voice |
| 8. Sensory System Symptoms | Blurred vision, hot and cold flushes, feelings of weakness, pricking sensation. |
| 9. Cardiovascular System Symptoms | Tachycardia, palpitations, pain in chest, throbbing of vessels, fainting feelings, missing heartbeats. |
| 10. Respiratory System Symptoms | Tightness in the chest, choking feelings, sighing, dyspnea. |
| 11. Gastrointestinal System Symptoms | Difficulty in swallowing, belching, dyspepsia (abdominal pain after eating, stomach burning, bloating, nausea, stomach fullness), bowel sounds, diarrhea, loss of weight, constipation. |
| 12. Genitourinary System Symptoms | Frequent urination, urgent urination, amenorrhea, frigidity, premature ejaculation, erectile dysfunction, impotence. |
| 13. Autonomic System Symptoms | Dry mouth, flushing, pallor, tendency to sweat, goosebumps, tension headache, raising of hair. |
| 14. Behavior at Interview | (1) General manifestations: nervous, restlessness, uneasy, biting fingers, clenching fists tightly, fumbling with handkerchief, facial muscle twitching, non-stopping feet, trembling hands, furrowed brow, facial tension, muscle tension, sigh-like breathing, facial pallor; (2) Physiological manifestations: swallowing, hiccups, fast resting heart rate, rapid breathing (over 20 breaths per minute), hyperreflexia, tremor, dilated pupils, eyelid twitching, easy sweating, exophthalmos. |

[Result analysis]

1. Total score: It can better reflect the severity of the disease. The Cooperative Group of the scale has compared the HAMA total score of 230 neurosis patients with different subtypes. The total score of neurasthenia was 21.00, anxiety was 29.25, depressive neurosis was 23.87; thus, anxiety symptoms were prominent among patients with anxiety. This group of patients had severe anxiety disorders.

2. Factor analysis: HAMA is only divided into two types of factor structures, physical factors and psychological factors. Somatic anxiety is consisted of the following seven items: (7) Somatic anxiety, muscular system; (8) Somatic anxiety: Sensory system; (9) Cardiovascular system symptoms; (10) Respiratory symptoms; (11) Gastrointestinal symptoms: (12) Genitourinary system symptoms; (13) Autonomic nervous system symptoms. Through factor analysis, it can not only specifically reflect the psychopathological characteristics of patients, but also reflect the treatment results of target symptom groups.

3. According to the information provided by the National Scale Cooperative Group, if the total score is more than 29 points, the patient may have severe anxiety; if it is more than 21 points, the patient must have obvious anxiety; if it is more than 14 points, the patient must have anxiety; if the score is less than 6 points, the patient has no anxiety symptoms. In general, the cut-off point for 14 HAMA items is 14 points.

[Application evaluation]

1. Reliability: After more than 10 systematic trainings, the consistency was excellent among the assessors. We have performed joint examinations on 19 patients with anxiety disorders. The consistency between the two assessors was quite good, the reliability coefficient r of the total score evaluation was 0.93, and the reliability coefficients of each individual symptom score were 0.83-1.00, and the P values were all less than 0.01.

2. Validity: The total score of HAMA can well reflect the severity of anxiety state. Our coefficient of severity of 36 cases of anxiety neurosis was 0.36 (P<0.05)

3. Practicability: The evaluation method of this scale is simple and easy, and can be used for anxiety disorders, but it is not suitable for estimating the anxiety state of various mental illnesses. At the same time, compared with HAMD, there are some repetitive items, such as depressive mood, somatic anxiety, gastrointestinal symptoms and insomnia, etc. Therefore, HAMA, like HAMD, cannot differentiate well between anxiety and depression.

Generally, it can be scored as follows: “1” symptoms are mild; “2” have definite symptoms, but do not affect life and activities; “3” symptoms are severe, need to be treated, or have affected life and activities; “4” symptoms are extremely severe, seriously affect their lives.

To assess the patient’s performance over the past week, please circle the score that best characterizes the patient.

REFERENCE

1. Hamilton M. The assessment of anxiety states by rating. Br J Med Psychol. 1959;32(1):50-5.
